# Dose rate and dose robustness for proton transmission FLASH-RT treatment in lung cancer

**DOI:** 10.3389/fonc.2022.970602

**Published:** 2022-08-15

**Authors:** Shouyi Wei, Haibo Lin, Sheng Huang, Chengyu Shi, Weijun Xiong, Huifang Zhai, Lei Hu, Gang Yu, Robert H. Press, Shaakir Hasan, Arpit M. Chhabra, J. Isabelle Choi, Charles B. Simone, Minglei Kang

**Affiliations:** ^1^ New York Proton Center, New York, NY, United States; ^2^ City of Hope, Orange County, Irvine, CA, United States

**Keywords:** proton FLASH-RT, ultra-high dose rate, pencil beam scanning, hypofractionation, lung stereotactic body radiation therapy, robustness, transmission proton beam

## Abstract

**Purposes:**

To evaluate the plan quality and robustness of both dose and dose rate of proton pencil beam scanning (PBS) transmission FLASH delivery in lung cancer treatment.

**Methods and materials:**

An in-house FLASH planning platform was used to optimize 10 lung cancer patients previously consecutively treated with proton stereotactic body radiation therapy (SBRT) to receive 3 and 5 transmission beams (Trx-3fds and Trx-5fds, respectively) to 34 Gy in a single fraction. Perturbation scenarios (n=12) for setup and range uncertainties (5 mm and 3.5%) were introduced, and dose-volume histogram and dose-rate-volume histogram bands were generated. Conventional proton SBRT clinical plans were used as a reference. RTOG 0915 dose metrics and 40 Gy/s dose rate coverage (V_40Gy/s_) were used to assess the dose and dose rate robustness.

**Results:**

Trx-5fds yields a comparable iCTV D_2%_ of 105.3%, whereas Trx-3fds resulted in inferior D_2%_ of 111.9% to the clinical SBRT plans with D_2%_ of 105.6% (p<0.05). Both Trx-5fds and Trx-3fds plans had slightly worse dose metrics to organs at risk than SBRT plans. Trx-5fds achieved superior dosimetry robustness for iCTV, esophagus, and spinal cord doses than both Trx-3fds and conventional SBRT plans. There was no significant difference in dose rate robustness for V_40Gy/s_ coverage between Trx-3fds and Trx-5fds. Dose rate distribution has similar distributions to the dose when perturbation exists.

**Conclusion:**

Transmission plans yield overall modestly inferior plan quality compared to the conventional proton SBRT plans but provide improved robustness and the potential for a toxicity-sparing FLASH effect. By using more beams (5- versus 3-field), both dose and dose rate robustness for transmission plans can be achieved.

## Introduction

Currently, in proton FLASH radiotherapy, single-energy transmission beams are the most feasible solution without any significant beamline modifications. In a cyclotron system, a very high beam current can be achieved by bypassing the energy degraders in the beamline, allowing for ultra-high dose rates and the potential for a FLASH effect (1–3). As proton beams shoot through the body instead of being stopped as in the conventional proton treatment plans, the dosimetric performances are also expected to be very different. Several studies have investigated proton pencil beam scanning (PBS) transmission planning in the lung (1, 2), head and neck (4, 5), brain (6), and liver patients (7, 8). All of those studies have shown promising dosimetric outcomes using transmission beams, which helps to inform the characteristics of proton transmission beams for treatment planning. The first human clinical trials, FAST-01 for bone metastases, used PBS single-beam to deliver 8 Gy/fraction for a group of 10 enrolled patients (9). The planned FAST-02 human trial for thoracic bone metastases will similarly use transmission beams, which are being considered in several future clinical trials (10). Thus, the robustness of dose and dose rate of transmission delivery are essential characteristics for making informative decisions regarding the FLASH effect.

Another critical aspect of FLASH transmission planning is evaluating FLASH dose rate coverage, especially in the organs at risk (OARs). Currently, there is no universal way of quantifying dose rate in proton PBS because it is unclear whether average or instantaneous dose rate is more relevant to the FLASH effect, and since the spot scanning procedure further confounds the dose rate definition. Previously, several groups have formed unique frameworks for studying the proton PBS dose rate in their transmission plans (2, 5, 11). Among the different methods, the average dose rate defined by the Folkerts et al. (11) is associated with time and is a relatively more conservative dose rate metric than others (2).

Lung cancer treatments have perhaps the highest toxicity of all sites treated and thus the most significant potential benefits from FLASH-RT (12–15). And the ultrafast dose delivery (beam time <1 s) may make the motion-related challenges less critical compared to conventional dose rate PBS (16). Previous in vivo FLASH experimental results showed evidence that FLASH-RT reduced the occurrence of lung fibrosis (17), spared lung progenitor cells, and limited the incidence of radio-induced senescence (18). Thus far, two studies have investigated the FLASH lung transmission plans (1, 2), both under stereotactic body radiation therapy (SBRT) dose fractionations. While these studies indicated current machine settings are capable of delivering transmission plans that achieve better quality than photon VMAT plan quality and high OAR FLASH dose rate coverage, they did not elucidate important planning considerations, especially in terms of the number of beams and beam arrangement, which may further affect the plan quality and FLASH dose rate coverage.

Moreover, plan robustness is critical in conventional proton RT plans using the Bragg peaks; however, as transmission plans use the plateau region that is least affected by range uncertainties, the plan robustness could become superior (16). Nevertheless, robustness in proton transmission FLASH plans has not been reported to date to our knowledge. This study aims to provide the first investigation to evaluate the sensitivity to the plan parameters, such as the number of beams and beam arrangement, as well as setup and range uncertainties, in terms of both dose and dose rate outcomes. Lastly, as dosimetric differences between single-energy FLASH transmission plans and conventional multi-energy intensity-modulated proton therapy (IMPT) plans have not been well characterized, we expect a comparison study might also provide a necessary reference to the future clinical practice for transmission FLASH planning. To achieve this goal, we optimized lung FLASH transmission plans with a varying number of beams and beam arrangements to assess the plan robustness for both dose and dose rate in this study. The conventional IMPT plans were used as references to better understand the plan quality of transmission planning.

## Methods and materials

### Treatment planning and dose rate quantification

The dose rate quantification was implemented into an in-house tool using the average dose rate (ADR) (2, 11), with a minimum spot time of 2 ms and a scanning speed of 10 mm/ms between adjacent spots. The beam delivery is presumed under a spot scanning mode, and the spot dwelling time and the scanning time between spots are both considered to calculate the averaged field dose rate. The details of the dose rate implementation and relations between beam currents and dose rates have previously been described (2).

Ten consecutive lung patients previously treated with proton SBRT at our institute were selected for this study which was approved by an internal research board (IRB). The tumor size ranged from 24.4-194.4 cm3, and the median size was 86.65 cm3. An iCTV was used to account for the respiratory motion based on the 4D CT images (2). A margin of 8 mm was isotropically extended from iCTV for PTV as a field target to place the spots for plan optimization. In transmission planning, only the flat region of a Bragg peak curve will be used to deposit doses to targets, and multiple-field optimization (MFO) with beams shooting through different angles is essential to achieve dosimetric conformity. The inverse planning system will provide the planning freedoms such as beam angles, spot weightings, spot placement, etc., to reach the planning goals and dose constraints. Thus, beam arrangement is an important factor in determining the plan quality and robustness. In theory, more fields with equal separations will help to improve the dosimetric distribution. In reality, the delivery efficiency will not allow an unlimited number of fields; according to the previous studies (2, 5, 6, 16, 19), 3-7 fields are sufficient to achieve optimal dose and dose rate metrics for target and OARs. Here, clinically possible beam arrangements with 5-field (Trx-5fds) and 3-field (Trx-3fds) beam arrangements were studied.

The transmission beams deliver dose via the plateau region rather than the Bragg peaks, the plan quality is less sensitive to range uncertainties and setup errors compared to the Bragg peak plans. Therefore, our current in-house transmission FLASH planning system includes MFO but not robustness optimization. The in-house planning platform based on the matRad framework uses a pencil beam convolution superposition (PCS) for dose calculation (20), and the dose grid was 1 mm. Eclipse treatment planning system used PCS version 16.1 as the dose calculation engine, and a 2.5 mm dose grid was used. The conventional multiple-energy MFO plans were generated using robust optimization in Eclipse, which uses worst-case scenario robust optimization (21). Twelve perturbation scenarios with a combination of 6 setup shifts of 5 mm in cardinal axes (± 5 mm) and ±3.5% CT HU to RSP (relative stopping power) conversion uncertainties were assessed (22). Twelve different dose distributions were calculated based on twelve perturbations. The objective function value for each iteration was calculated based on the worst-case dose result. Conventional multiple-energy proton IMPT plans planned by professional dosimetrists and reviewed by radiation oncologists served as references to the transmission FLASH plans. Following our current clinical practice, the global maximum dose is less than 115% of the prescribed doses for both FLASH and conventional plans.

### Plan quality, dose rate, and robustness analysis

Dosimetric performances were evaluated following the RTOG 0915 guidelines for lung treatment planning. The dosimetric metrics of CTV D2%, spinal cord D1.2cc, D0.35 cc, Dmax, lung-GTV V7Gy, V7.4Gy, and heart D15cc, Dmax were evaluated. The V40Gy/s was used to quantify the dose rate performance for FLASH plans, which indicates the voxels ratio that received doses with a dose rate > 40 Gy/s in OARs and target structures. A dose rate volume histogram (DRVH) was also used to evaluate the dose rate distribution vs. volume for a region of interest (2).

Similarly, a clinically applied robustness evaluation method was implemented for dose and dose rate robustness analysis based on the twelve perturbation scenarios. The nominal plans were normalized to 100% prescribed dose covering 95% target volume. The conventional second worst-case and median scenarios for the D95% were employed to evaluate plan robustness. The max-min dose distribution is the maximum dose differences, i.e., maximum minus minimum dose values, among the 12 perturbations from the nominal plans. To visualize the dose rate volume histograms with uncertainty bands, the bandwidth (BW) of a dose rate metric was used to evaluate the robustness, with an example shown below:


(1) $${\text{BW}}_{{\text{V}}_{40\text{Gy}/\text{s}}}\;={\text{V}}_{40\text{Gy}/\text{s},\text{ upper bound}}-{\text{V}}_{40\text{Gy}/\text{s},\text{ lower bound}}$$

Here the bandwidth of V40Gy/s is given by the difference between the upper and lower bounds of V40Gy/s from the 12 perturbation scenarios of DRVHs. Similar to the max-min dose distribution, the max-min dose rate distribution was also calculated to identify the locations where the dose rate has the most uncertainties with perturbation existing.

## Results

### Plan quality assessment - dosimetric performances

**Figure 1:**
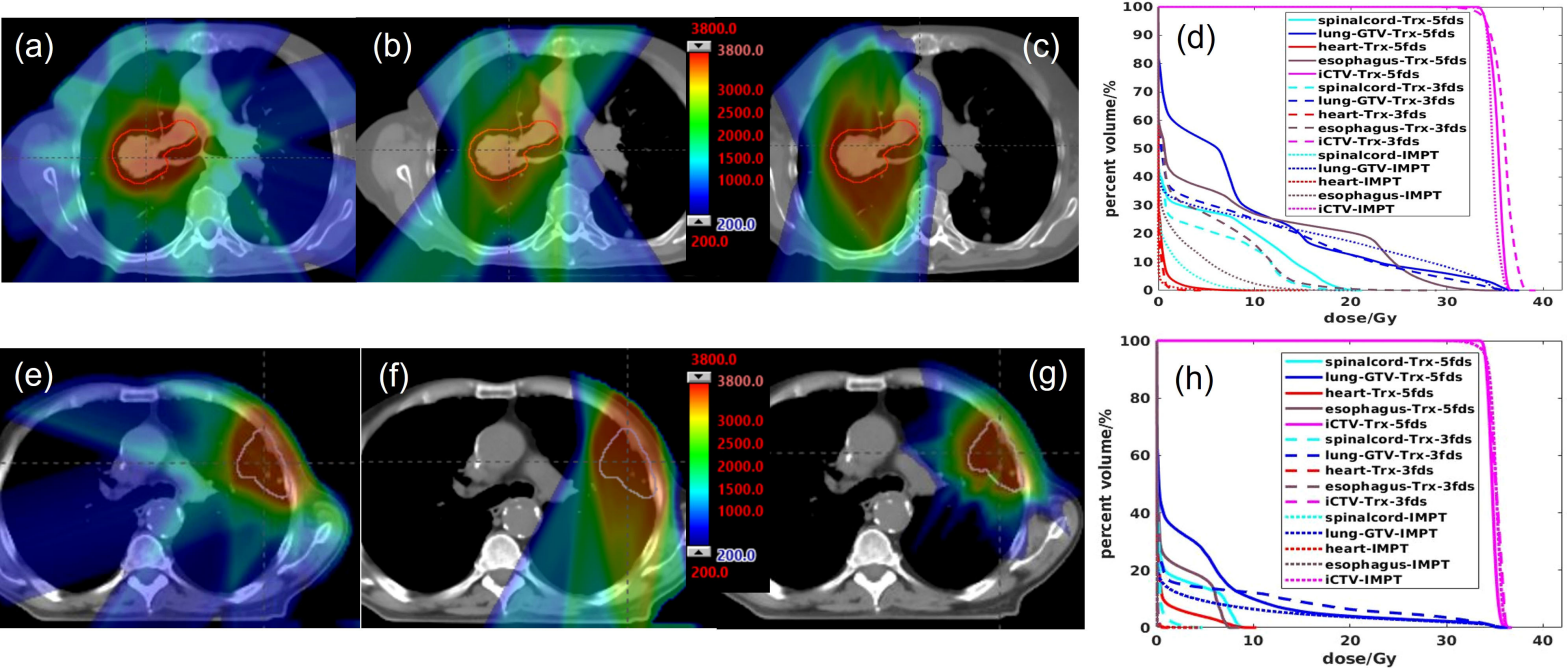
The 2D dose distributions and DVH comparisons for 2 selected patients. **(A, E)** Trx-5fds plan 2D dose distribution, **(B, F)** Trx-3fds plan 2D dose distribution, **(C, G)** conventional PBS plan dose distribution, **(D, H)** DVHs of the Trx-5fds, Trx-3fds, and conventional IMPT-SBRT plans.

Two patients were selected to demonstrate the differences in the treatment plans of the transmission and multiple-energy PBS plans in terms of dose distribution and DVHs (**Figure 1**). Patient #1 has a tumor located on the right mid lung, and the results are shown in **Figures 1A–D**, and the 2D dose distributions for different types of plans are visually very different. As displayed in **Figures 1A, B** a transmission beam can completely shoot through the body, the Bragg peak is outside of the patient, and all of the normal tissue and OARs in the beam path receive similar doses to the target for a given beam. Multiple-field shooting from different angles can overlap at the target to give the target the full prescribed doses and to increase conformality. In contrast, the OARs beyond the target only receive a relatively small fraction of the prescribed dose. Compared to conventional multiple-energy proton PBS plans, transmission plans generate dose spillage to the heart and healthy lung tissues beyond the target in the beam paths. As the Trx-5fds plans used equal beam separation of 72 degrees, the dose spillage is spread equally to the body. While in the Trx-3fds, less low dose spillage to the right lung is achieved compared to the 5-field arrangement as displayed in the DVHs. However, as the prescription doses are the same, the average dose per beam in the Trx-3fds plans tends to be higher than that of the Trx-5fds plans, which increases the doses in healthy tissues irradiated by the Trx-3fds beams. In contrast, the conventional IMPT plans use multiple-energy Bragg peaks to deliver conformable doses to the target, and the dose spillage to normal tissues is much less than transmission plans. Similar planning outcomes can be observed for patient #2 (**Figures 1E–H**), whose tumor is located in the left anterior lung close to the chest wall. Given the tumor’s location, 3 beams deliver less dose to normal tissues and can completely spare the contralateral lung relatively to 5-field plans. The 5-beam plan, however, splits the doses to a large area of tissue and achieves better target conformity.

The statistics of the dose performances of the 10 lung patients are summarized in **Table 1**. All dose metrics were averaged for treatment plans of Trx-3fds, Trx-5fds, and conventional PBS plans, respectively. Trx-5fds yields comparable target uniformity, while Trx-3fds results in inferior (p<0.05) target uniformity compared to the conventional IMPT plans. Trx-3fds achieve improved results in most OARs dose metrics, including the spinal cord, lung-GTV, and esophagus, than Trx-5fds. The dose metrics of the heart are comparable using 3- and 5-field arrangements in FLASH planning. Trx-5fds results in worse dose performances in all OARs except for the heart than the IMPT plans. While still modestly inferior, the Trx-3fds dose metrics are improved to be closer to the IMPT plans relative to the Trx-5fds dose metrics.

**Table 1:** Summary of dose metrics of RT structures in Trx-5fds, Trx-3fds and IMPT plans.

RT Structure	Dose metric	Trx-5fds	Trx-3fds	IMPT-SBRT
iCTV	D_2%_(%)	105.3 (2.1)	111.9 (7.9) *	105.6 (1.9)
Spinal cord	D_1.2cc_(Gy)	16.2 (5.7)**	12.4 (6.1)*	9.7 (6.0)
	D_0.35 cc_(Gy)	18.1 (5.6)**	14.8 (5.2)	11.6 (6.5)
	D_max_(Gy)	19.2 (6.2)*	16.8 (6.3)*	13.7 (6.9)
Lung-GTV	V_7Gy_(%)	27.5 (12.2)**	20.4 (11.6)**	14.2 (8.0)
	V_7.4Gy_(%)	24.4 (11.4)**	20.0 (11.4)*	13.9 (7.9)
Heart	D_15cc_(Gy)	14.6 (13.7)	13.5 (12.5)	16.8 (15.2)
	D_max_(Gy)	20.2 (13.9)	20.6 (16.9)	23.7 (14.6)
Esophagus	D_5cc_(Gy)	17.8 (10.1)**	12.6 (10.9)	10.1 (14.3)
	D_max_(Gy)	23.4 (10.5)*	21.3 (13.8)**	18.4 (13.9)

*Denotes p<0.05, ** denotes p<0.01, two-tailed student t-test between transmission and conventional IMPT plans

### Plan quality assessment - dose rate performances

**Figure 2:**
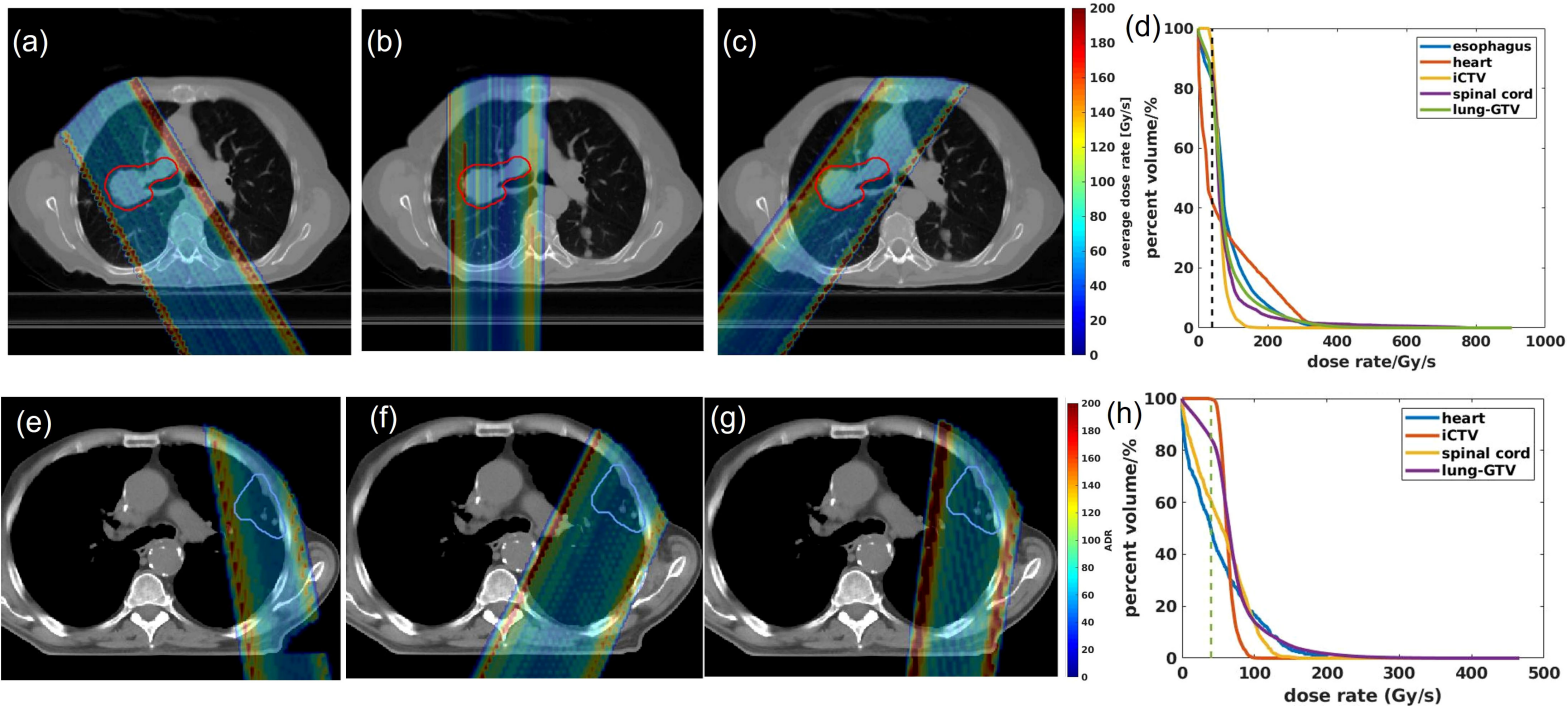
**(A–C)** Dose rate distributions for beam 1, 2 and 3 for patient #1, **(D)** DRVHs of the Trx-3fds plan, **(E–G)** dose rate distributions for beam 1, 2 and 3 for patient #2, **(H)** DRVHs for the Trx-3fds plan.

**Figure 3:**
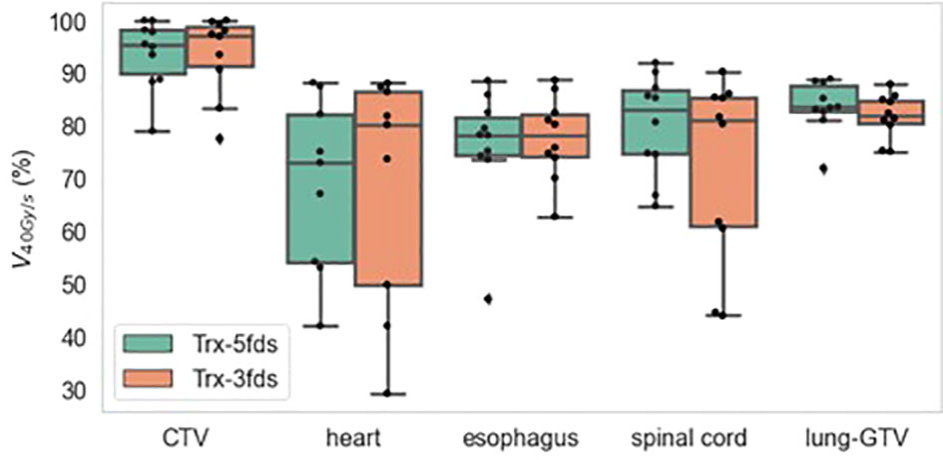
V_40Gy/s_ statistics of 10 patients for target and OARs. The dots represent each patient, the line inside the box represents the mean value, and the error bars outside the box represent the 25-75^th^ percentile of the scattered values.

The 2D averaged dose rate distribution, and DRVHs of Trx-3fds plans for the two lung patients’ plans are shown in **Figures 2A–H**. The field edges give higher dose rate distributions consistent with the findings in (2, 11) for both patient cases. The streak-like patterns in the dose rate distributions also reflect the PBS spot scanning patterns for the ADR calculation methods, consistent with (2, 11). For the DRVHs, as observed for patient #1, the FLASH coverage can be as high as 80% for most ROIs, as the tumor location is close to the spinal cord and esophagus, which receive very high exit dose with high FLASH dose rate coverage. For patient #2, the FLASH coverages in the heart and spinal cord drop, reflecting that only the field penumbrae cover such RT structures. Although they correspond to a large dose rate at the edges, most volume beyond the hot edges into the penumbrae falls to lower dose rate values, causing the overall drop in the FLASH coverages. The statistics of the V40Gy/s of the 10 lung patients are summarized in **Figure 3**. Except for the spinal cord, the differences in V40Gy/s in all the other RT structures are comparable between the Trx-5fds and Trx-3fds cases.

### Plan does robustness assessment

**Figure 4:**
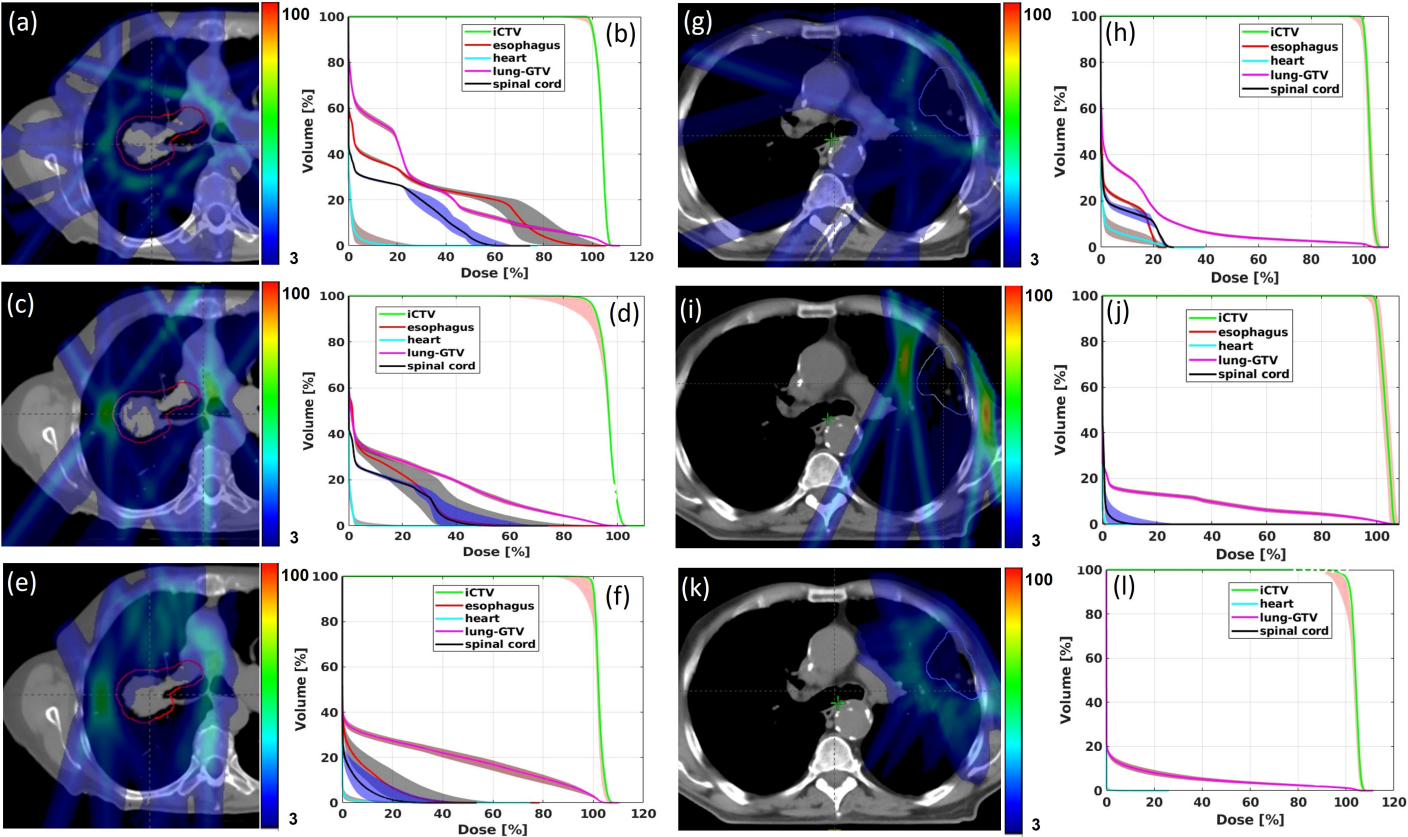
Max-min dose distributions from 12 perturbations for Trx-5fds **(A, G)**, Trx-3fds **(C, I)** and conventional PBS plans **(E)** and **(K)** for patient 1 and 2. DVH bands from 12 perturbations for Trx-5fds **(B, H)**, Trx-3fds **(D, J)** and conventional IMPT plans **(F, L)**.

The max-min dose distributions between the perturbation plans and the initial plan were calculated for Trx-5fds, Trx-3fds, and conventional IMPT plans, as well as the DVH bands for each plan, are shown in **Figure 4** for the 2 selected patients. As the transmission plans place Bragg peaks outside of the patient’s body, most of the uncertainty in the dose distribution arises from the edge of the fields, especially at the rim of the target where the fields overlap. The max-min uncertainties distribute more evenly around targets in 5-field plans than 3-field plans. While in conventional IMPT plans, as Bragg peaks are used to deliver doses to the targets, the uncertainty occurs at the lateral field edge and the distal edges. We can also observe from these two patient cases that the robustness achieved with Trx-5fds is better than Trx-3fds and conventional PBS plans in the target DVH bands (**Figures 4B, D, F, H, J, L**), with narrower CTV bands indicated.

**Figure 5:**
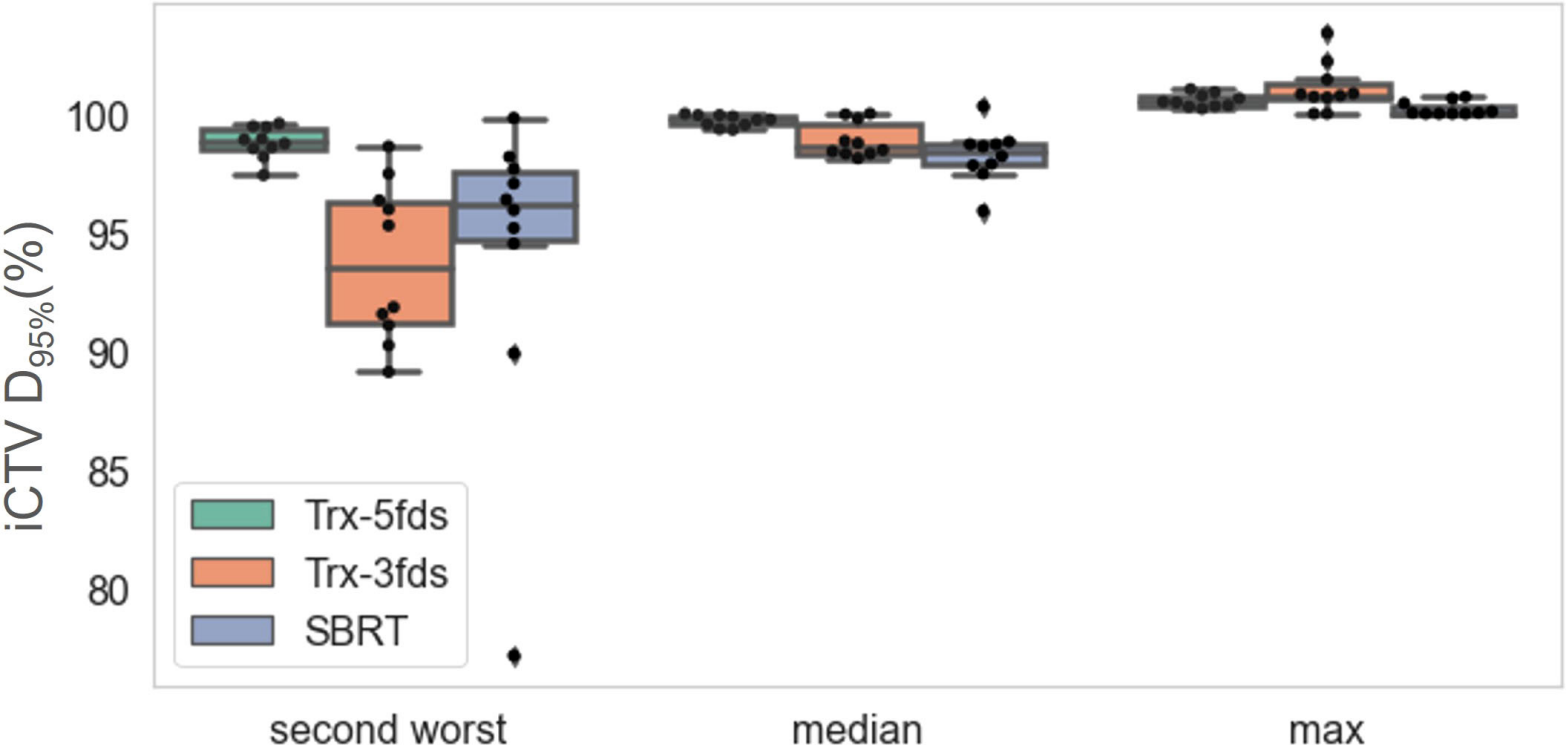
The D_95%_ of iCTV statistics distributions for the 10 patients for the second-worst cases, median and max values. The dots represent each patient, the line inside the box represents the mean value, and the error bars outside the box represent the 25-75^th^ percentile of the scattered values.

The second worst-case scenario, media, and max of D95% were investigated as indicators for the robustness assessment. The statistics of the CTV D95% of the different plans for the 10 patients are presented in **Figure 5**. Trx-5fds result in the narrowest distribution with the least CTV D95% variation compared to the other two types of plans. Trx-3fds result in a worse distribution in D95% for the perturbations than conventional IMPT plans in general but are presented with fewer outliners in this case. The better robustness performance of Trx-5fds can benefit from the beam arrangement and number of beams, i.e., 5 fields are evenly distributed 72 degrees apart, thereby minimizing the setup errors effect in different directions. As Trx-3fds have fewer fields and the angles are optimized to avoid lung, heart, and other critical organs, the uncertainties from setup errors become more significant than those of Trx-5fds. For the median and max of D95%, all 3 types of plans achieve relatively comparable results.

### Plan dose rate robustness evaluation

**Figure 6:**
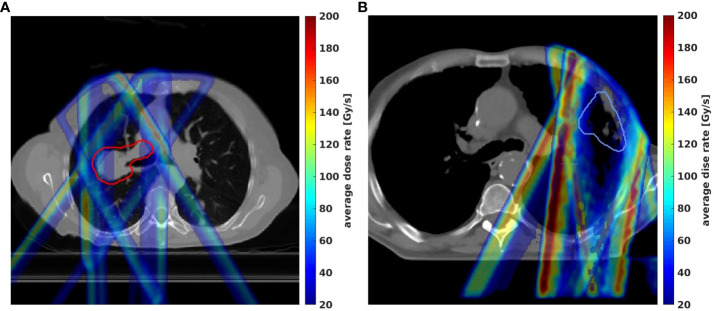
Max-min dose rate distributions in 2D from 12 different perturbation scenarios for lung patient #1 **(A)** and patient #2 **(B)** were demonstrated by the Trx-3fds plans.

Similar to the max dose uncertainties distribution, the max dose rate distributions between the perturbation plans and the initial plan were calculated for Trx-5fds and Trx-3fds. As shown in **Figure 6**, the largest uncertainty in dose rate from the 12 perturbation scenarios arising from the field edges, which has a similar pattern to the dose case as shown in **Figure 4**.

**Figure 7:**
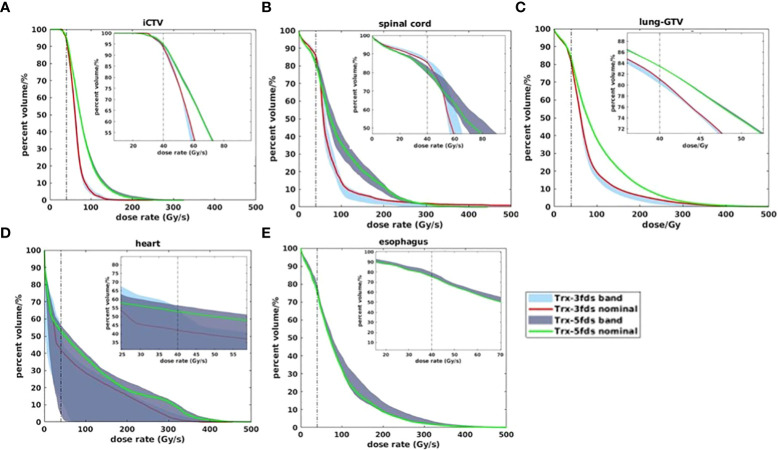
DRVH bands for different RT structures for Trx-3fds and Trx 5fds for patient #1, target iCTV **(A)** and OARs spinal cord, lung-GTV, heart, and esophagus **(B–E)**.

**Figure 8:**
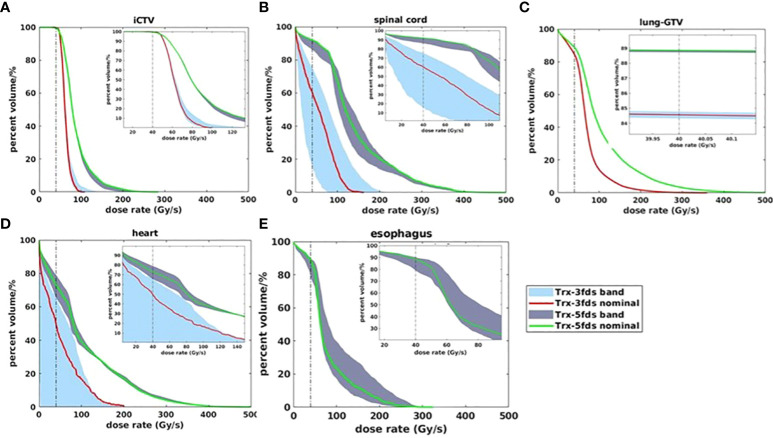
DRVH bands for different RT structures for Trx-3fds and Trx 5fds for patient #2, target iCTV **(A)** and OARs spinal cord, lung-GTV, heart, and esophagus **(B–E)**.

The DRVH bands for each OARs are shown in **Figures 7**, **8** for 2 selected patients. The iCTV and lung-GTV bands are very narrow for both patients, with bandwidths of V40Gy/s less than 2%. Large variations from perturbations can be consistently observed in the spinal cord and heart for both 3-field and 5-field transmission plans, partially covered by the transmission beams in the penumbra regions. Using 3-field can completely avoid the esophagus, thus, there are no DRVHs for 3-field plans shown in **Figures 7E**, **8E**. Another significant difference between the 2 patients can be observed from the spinal cord DRVH for 3-field transmission plans. Patient #1 has a much narrower dose rate uncertainty in the spinal cord than that patient #2. This can be explained as the main portion of beams in patient #1 shooting through the spinal cord while the penumbra regions of the beams shoot through the spinal cord for patient #2.

**Figure 9:**
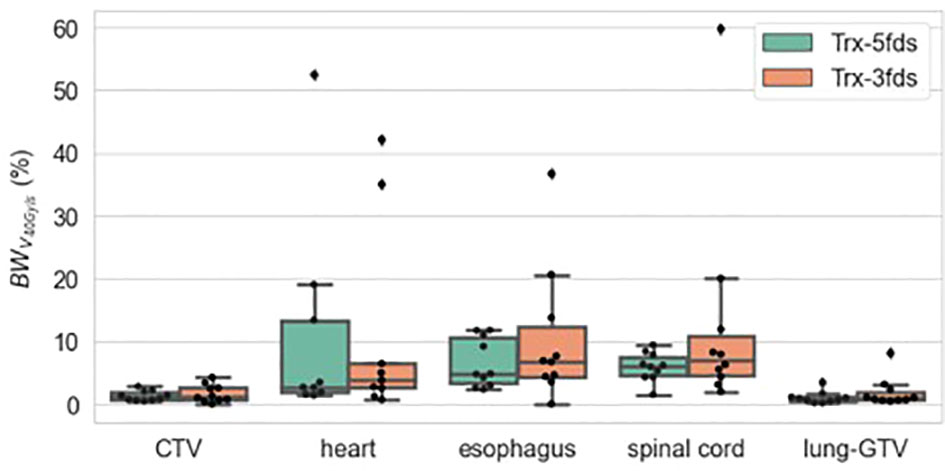
V_40Gy/s_ robustness of 10 patients of different RT structures. The dots represent each of the 10 patients, the line inside the box represents the mean value, and the error bars outside and the box represent the 25-75^th^ percentile of the scattered values.

**Figure 9** is the V40Gy/s robustness of 10 patients of different RT structures. The V40Gy/s BWs for iCTV and lung-GTV for both Trx-3fds and Trx-5fds plans are < 2% on average, although those of Trx-5fds plans appear to be slightly narrower. V40Gy/s BWs vary differently for the spinal cord, esophagus, and heart, which tend to have more considerable dose rate variations when perturbations exist. This phenomenon is mainly due to the relative location of these OARs to the target, causing it to be in the middle of beam paths or penumbrae. In general, Trx-5fds appears to be slightly more dose rate robust than Trx-3fds, with ~1%, 2%, and 5% on average in the heart, spinal cord, and esophagus, respectively.

## Discussion

This study simulates realistic planning settings in FLASH treatment planning characterized with feasibility in machine delivery based on beam current, energy, and minimum MU/spot, and practical planning considerations characterized by practical dose prescription and beam arrangement, including number and angles. We further assessed different plan quality regarding dose and dose rate performances as well as robustness in comparison with conventional IMPT plans using Bragg peaks with multiple energy layers.

The in-house planning platform allows inverse planning and, therefore, improves the transmission plans’ quality given the OAR doses and target uniformity. Despite the FLASH effect, which is currently the most standard approach, this study still considers the dose metrics imposed by conventional dose rate plans for the transmission plans. While this could be subject to changes in the future as more biological or clinical trial evidence of FLASH is to be uncovered, right now, assuming the same dose constraints may enable us to evaluate the “lower bound” of the FLASH plans’ quality, providing benchmarks for future clinical planning practices.

Another significant and novel contribution of this work is that we evaluated the robustness of the transmission plans in terms of both dose and dose rate. Previously, van Marlen et al.’s study (1) indicated that transmission plans using plateau regions do not involve the range uncertainties as in conventional Bragg peak-based plans and, therefore, can achieve better robustness. Our results show that with 5 transmission beams, better plan robustness can be achieved in target coverage. Still, with 3 beams, such robustness can be degraded to a similar or slightly worse level than the conventional IMPT. One thing to note is that our current in-house FLASH planning system does not include robustness optimization, once implemented, which may further improve the robustness of transmission plans. The value of robustness optimization in FLASH planning requires further investigation. For dose rate robustness, the uncertainty of setup and range perturbations in V40Gy/s is especially small (< 3% for lung-GTV). The results vary for other OARs such as the esophagus, heart, and spinal cord, given their relative anatomical location to the target. For example, we expect the larger differences in the heart, spinal cord, and esophagus since we tend to spare these OARs with the beam penumbras that may have larger dose gradients. Such penumbra regions also correspond to lower doses that may not be able to trigger the FLASH effect, given the recent biological observations that a dose threshold > 5 Gy may need to be met (23–27). The lack of dose rate robustness and low dose deposition will require careful thought in future treatment planning practice for FLASH, especially when OARs are present in such regions. Beam numbers and angles are critical parameters for treatment planning optimization, which exert a crucial impact on the planning outcomes. This study indicates that more transmission beams might reduce the sensitivity to setup errors to achieve a better dose rate robustness. Due to the nature of the transmission beams, when more beams are used to deliver target doses, the low dose spillage may be increased to OARs, and the tradeoff between dose rate robustness and low dose spillage should be balanced in designing transmission FLASH plans.

Similar to the photon beams, the transmission FLASH always has the exit dose. PBS field employs scanning spots to deliver the prescribed dose. The depth dose of a broad PBS field is very different from the photon beam that follows an exponential attenuation beyond the buildup region. The depth dose of the plateau region slightly increases with depth at the first 30 cm in water for a 250 MeV proton beam. Considering the beam divergence and scattering effect, the entrance dose of the proton beam is very close to that of the exit dose. In contrast, the entrance dose is always much higher than the exit dose beyond the maximum dose depth for a photon beam.

We chose the same minimum MU/spot of 400 for both Trx-5fds and Trx-3fds plans, which resulted in around 80% OARs FLASH coverage in most cases. The ADR was used for quantifying the FLASH dose rate coverage in our treatment plans, which is a conservative definition in proton PBS planning (2). Previous studies also demonstrated that when using ADR, the FLASH sparing effect can be observed in the skin of mice at a dose rate of ~ 65 Gy/s (28). The minimum MU/spot choice could be flexible given the machine’s delivery limit, especially the beam current and allowed minimum spot time. Here we chose a feasible cyclotron current for the Varian ProBeam system, and under this current, the FLASH coverage for both plans is close. We demonstrated earlier in (7, 8) that a less minimum spot time in the FLASH spot map delivery will allow smaller MUs in the treatment plans at the fixed beam current, likely resulting in superior plan quality to ones with greater minimum spot times.

Motion is an important factor for lung treatment plans, especially given the large fractionation doses in SBRT (29–31). For FLASH plans, as the delivery of the spot maps is reduced to be less than one second, the motion within a fraction is minimal, but an accurate coverage of the dose in the target requires timely monitoring of the phase of the motion, thereby posing a particular challenge in FLASH delivery for moving targets. Nevertheless, the time intervals between beams are not trivial, giving rise to motion-related uncertainties in the treatment. Our study used iCTV methods to generate a more accurate target volume for treatment planning. However, the interplay effect was not included in the treatment planning study. Multiple methods have been mentioned to reduce the motion-caused interplay effect, such as using an abdominal compression belt (32) or deep inspiration breath-hold (DIBH) (33, 34). To what extent the interplay will affect the dose and dose rate accuracy warrant more detailed investigation.

Our work has demonstrated the dosimetric advantages of clinical IMPT plans over transmission plans, with superior OAR doses, comparable target uniformity, and slightly inferior plan robustness in target coverage. Significant efforts have been made to bring the Bragg-peak-based plans for FLASH applications (16, 35), including the Spread-out of Bragg peak (SOBP) and single-energy Bragg-peak plans. While this may present new technical challenges in FLASH, we expect such approaches will fill the gaps between the transmission plans and clinical IMPT plans for better FLASH treatment outcomes.

## Conclusion

Using the highest energy from the cyclotron system to deliver FLASH-RT is practical for clinical application. The MFO inversed optimization can achieve quality treatment plans for transmission beams. Transmission plans yield overall modestly inferior plan quality compared to the conventional proton SBRT plans but provide improved robustness with 5 beams while allowing for the potential for achieving a FLASH effect in this lung cancer study. By introducing more beams (5- versus 3-field), both dose and dose rate robustness for the transmission plans can be achieved.

## Data availability statement

The raw data supporting the conclusions of this article will be made available by the authors, without undue reservation.

## Ethics statement

The studies involving human participants were reviewed and approved by Western IRB. Written informed consent for participation was not required for this study in accordance with the national legislation and the institutional requirements.

## Author contributions

Conceptualization, MK, SW, CBSII, and HL; methodology, MK and SW, software, SW; validation, MK and SW; formal analysis, SW; investigation, MK and SW; resources, JIC, CBSII, and HL; data curation, MK and HL; writing—original draft preparation, SW and MK; writing—review and editing, all authors.; visualization, MK and SW; supervision, CBSII and HL; project administration, HL; funding acquisition, HL. All authors have read and agreed to the published version of the manuscript.

## Funding

The authors declare that this study received funding from the Varian Research Grant. The funder was not involved in the study design, collection, analysis, interpretation of data, the writing of this article or the decision to submit it for publication.

## Acknowledgments

The authors would like to thank Dr. Michael Folkerts and Krystian Wrobel for valuable discussions and suggestions. This study was partly supported by Varian, a Siemens Healthineers Company.

## Conflict of interest

HL, JIC, and CBSII report an honorarium from Varian Medical Systems. This study was partly funded by Varian, a Siemens Healthineers Company.

The remaining authors declare that the research was conducted in the absence of any commercial or financial relationships that could be construed as a potential conflict of interest.

## Publisher’s note

All claims expressed in this article are solely those of the authors and do not necessarily represent those of their affiliated organizations, or those of the publisher, the editors and the reviewers. Any product that may be evaluated in this article, or claim that may be made by its manufacturer, is not guaranteed or endorsed by the publisher.
